# Continuous transport of Pacific-derived anthropogenic radionuclides towards the Indian Ocean

**DOI:** 10.1038/srep44679

**Published:** 2017-03-17

**Authors:** Daniela Pittauer, Stephen G. Tims, Michaela B. Froehlich, L. Keith Fifield, Anton Wallner, Steven D. McNeil, Helmut W. Fischer

**Affiliations:** 1 University of Bremen, MARUM - Center for Marine Environmental Sciences, Bremen, 28359, Germany; 2 University of Bremen, Institute of Environmental Physics, Bremen, 28359, Germany; 3 Australian National University, Department of Nuclear Physics, Canberra, ACT 2601, Australia

## Abstract

Unusually high concentrations of americium and plutonium have been observed in a sediment core collected from the eastern Lombok Basin between Sumba and Sumbawa Islands in the Indonesian Archipelago. Gamma spectrometry and accelerator mass spectrometry data together with radiometric dating of the core provide a high-resolution record of ongoing deposition of anthropogenic radionuclides. A plutonium signature characteristic of the Pacific Proving Grounds (PPG) dominates in the first two decades after the start of the high yield atmospheric tests in 1950’s. Approximately 40–70% of plutonium at this site in the post 1970 period originates from the PPG. This sediment record of transuranic isotopes deposition over the last 55 years provides evidence for the continuous long-distance transport of particle-reactive radionuclides from the Pacific Ocean towards the Indian Ocean.

Plutonium isotopes present in today’s oceans originate from various sources^1^,^2^,^3^. The major portion can be attributed to nuclear weapons testing in the atmosphere between 1952 and 1980. The most abundant plutonium isotopes in the marine environment are ^239^Pu (T_1/2_ = 24,110 yr) and ^240^Pu (T_1/2_ = 6,561 yr). The short-lived ^241^Pu (T_1/2_ = 14 yr) has in large part decayed, and its progeny ^241^Am (T_1/2_ = 433 yr) is now another prominent isotope associated with nuclear fallout.

In the 1950’s a series of nuclear tests was performed by the United States in the Marshall Islands, namely at the Bikini and Enewetak Atolls, an area known as the Pacific Proving Grounds (PPG). These tests released substantial quantities of radioactive material into the environment. Between 1946–1958 the total estimated fission yield from the PPG tests was 57.7 Mt, which corresponds to over 30% of the estimated 189 Mt total global fission yield from all atmospheric tests^4^. The distinct ^240^Pu/^239^Pu atom ratio provides a fingerprint of PPG-derived material^5^, and ratios in the sediments of the Northwest Pacific indicate a notable contribution from the PPG^6^,^7^. To this day significant amounts of plutonium continue to be released from the PPG into the surrounding waters. Ocean water from the test area is transported by the North Equatorial Current (NEC) towards the Philippines and further north with the Kuroshio Current. Indeed, coral records from the Pacific island Guam, ~2,000 km west of PPG and along the NEC pathway, show spikes in the ^14^C that can be linked to individual PPG tests^8^. It has been known for several years that plutonium from the PPG can be found in the Northwest Pacific marginal seas, particularly in the East China Sea^9^,^10^,^11^ and along the Japanese west coast^12^. Detectable PPG signals are also reported in sediments from the Sulu and South China Seas^13^,^14^.

Part of NEC water also heads south with the Mindanao Current and further on via the Indonesian Throughflow (ITF, Fig. 1). Coral-based ^14^C records from the Makassar Strait area suggest that a large proportion of Pacific-derived radiouclides is being transported southwards^15^. Surface water samples from the Sulu Sea and the Makassar Strait also showed a PPG plutonium contribution^16^. The significance of any PPG derived plutonium in ITF sediments is not presently known, however high ^241^Am concentrations were recently reported in ITF sediments from the Lombok Basin off Sumba Island, Indonesia^17^. Gamma spectrometric analysis demonstrated the sediment core contained a high inventory of ^241^Am. The aims of this study, performed on a sediment core taken at the same Lombok Basin station^17^, were to

1. determine, if high ^241^Am concentrations at the Lombok Basin site are correlated with similarly high concentrations of plutonium isotopes, providing definitive evidence of transport of the transuranic radioisotopes through the ITF,
2. ascertain the fraction of PPG derived plutonium based on known end-members from the PPG and global weapons fallout, and
3. evaluate the depositional histories of the anthropogenic isotopes.

The available 50 cm long deep sea sediment multicorer (MUC) core, GeoB 10065-9 MUC-B, covering the Anthropocene period with high resolution, was recognized as a potentially suitable material for achieving these aims. Notably, the sampling site is located in a region affected by seasonal coastal upwelling during the southeast summer monsoon. Core sediments consist of olive to dark olive foraminiferral mud, comprising mainly of a lithogenic portion (73%)^18^. The surface sediment consist largely of clay minerals^19^ and CaCO_3_ (16%)^18^. Total organic carbon (C_*org*_) and nitrogen (N) are high at this site (2.3% and 0.31%, respectively) owing to the upwelling-related high primary productivity. C/N ratios of 7.5 and *δ*^13^*C* of −20.3 point to a predominantly marine origin of deposited organic matter^18^.

## Results

### Age model and sedimentation rates

**Figure 2:**
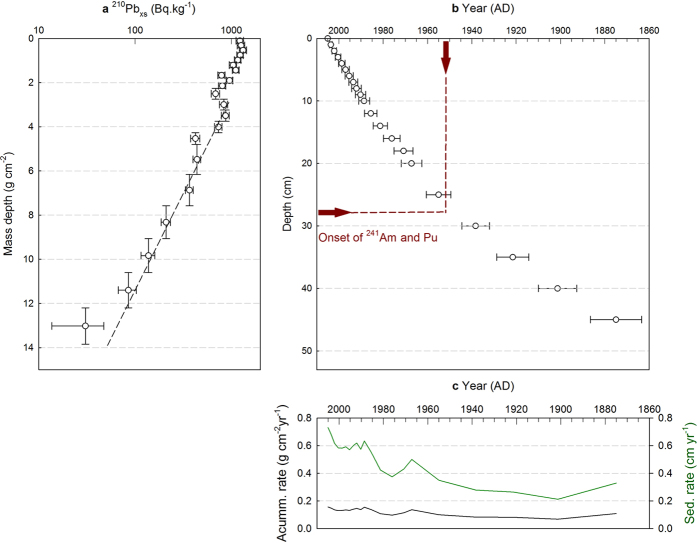
Chronology. (**a**) Depth profile of excess ^210^Pb activity in dry mass measured by gamma spectrometry in the GeoB 10065–9 MUC-B core^17^ plotted at mass depth scale for compaction correction. Horizontal error bars reflect combined uncertainties (1 *σ*), including counting statistics and detector calibration uncertainty. Vertical error bars represent the thickness of the depth increment. An exponential fit, depicted as a dashed line, was used for estimation of the residual inventory below 50 cm depth, required for the application of the Constant Rate of Supply (CRS) age model. (**b**) Chronology of the MUC-B core based on the CRS model. Horizontal error bars represent model age uncertainties (1 *σ*). The onset of ^241^Am and ^239^Pu (1952) is indicated by arrows. (**c**) Sedimentation and accumulation rates derived from the chronology.

The depth profile of the naturally occurring ^210^Pb activity concentrations (Fig. 2a) reflects a very high sedimentation rate at the study site. The sediment core chronology based on the excess ^210^Pb, developed within this study, is presented in Fig. 2b. The first appearance of ^241^Am and ^239^Pu, expected around 1952, appears at a depth of ~27.5 cm, in an excellent agreement with the ^210^Pb age model. The average sedimentation rates vary between 0.61 ± 0.02 cm ·  yr^−1^ in the top layers and 0.27 ± 0.06 cm · yr^−1^ towards the bottom of the core, which covers approximately the last 160 years. These values correspond to mass accumulation rates of 0.14 ± 0.01 and 0.09 ± 0.02 g · cm^−2^ · yr^−1^. These high sedimentation rates are similar to those determined at this site over the last ~6,000 years from the parallel gravity core GeoB 10065–7 which, based on the radiocarbon record of mixed layer and thermocline dwelling planktonic foraminifera, gave sedimentation rates between 0.11 ± 0.05 and 0.35 ± 0.05 cm · yr^−1 ^17.

### Anthropogenic isotopes

**Figure 3:**
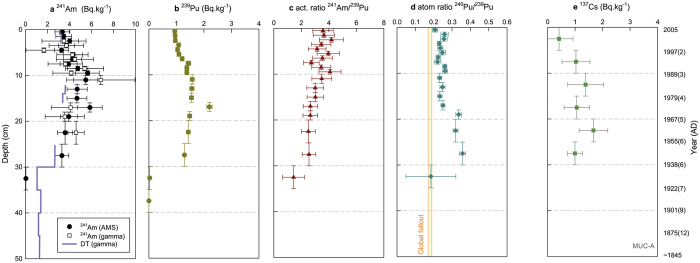
Depth profiles of anthropogenic radionuclide activity concentrations. (**a**) ^241^Am measured by gamma spectrometry^17^ (reference date: October 2010; for samples in which no Am was detected, decision thresholds (DT) are plotted instead) and by AMS (reference date: July 2015). (**b**) ^239^Pu measured by AMS. (**c**) ^241^Am/^239^Pu activity ratios (calculated from the AMS dataset). (**d**) ^240^Pu/^239^Pu atom ratios. (**e**) ^137^Cs measured by gamma spectrometry in the parallel core MUC-A^17^ decay corrected to the sampling date (September 2005). Vertical error bars represent the thickness of the depth increment. Horizontal error bars represent combined counting and calibration uncertainties (1 *σ*) for gamma spectrometry data and combined statistical and systematic errors (1 *σ*) for AMS data. The right axis shows ages derived from the CRS age model.

Gamma spectra from the uppermost samples of the core show high concentrations of ^241^Am. The detection of this radionuclide by gamma spectrometry is difficult, owing to the rather low gamma-ray energy (59.5 keV) and moderate emission probability, but for these samples evidence for ^241^Am was clearly above the decision threshold of 1–5 Bq · kg^−1^ for the experimental setup used. The threshold depends mainly on the sample mass, measurement time and the activities of other gamma emitters in the sample and detector system. Comparable activities with lower experimental uncertainty were confirmed within this study by means of accelerator mass spectrometry (AMS) and are plotted in Fig. 3a. Clear evidence for ^137^Cs was not present in the individual gamma spectra (<1.5 Bq · kg^−1^), but had been quantified in the parallel core MUC-A by summing up gamma spectra of 5 successive 1 cm depth intervals (Fig. 3e)^17^.

Plutonium-239 activity concentrations determined in sediment layers by AMS are plotted in Fig. 3b. The isotope was detected in the top 30 cm of the core, with high activity concentrations, comparable to those from deep ocean sediments between the major test sites of the PPG^6^. Below this depth traces of Pu were found, these however are almost two orders of magnitude lower than activities above 30 cm and are probably the result of natural replacement (e.g., due to biological or physical mixing) or reflect displacement of material during sampling. These values were therefore not considered for further interpretation. A broad maximum in the ^239^Pu concentration at approximately 10–20 cm depth can be observed. The activity is slightly lower in the top 10 cm.

Americium-241 shows a qualitatively similar depth profile (Fig. 3a). The Spearman’s rank-order correlation was used to determine the relationship between ^241^Am (AMS derived dataset) and ^239^Pu activities and yielded a statistically significant (rs(18) = 0.707, p = 0.001) positive correlation.

The ^240^Pu/^239^Pu atom ratio (Fig. 3d) varies between 0.20 at the top to 0.36 at 30 cm depth, and a single measurements of the ^241^Pu/^239^Pu ratio at 18–20 cm depth yields an atom ratio of (1.72 ± 0.20) · 10^−3^ (July 2015, equivalent to (2.77 ± 0.32) · 10^−3^ at September 2005). ^236^U content in all investigated sediment samples was below our detection limit.

## Discussion

The calculated inventories (Eq. 1) of the particle reactive radionuclides studied are much higher than would be expected from atmospheric fallout deposition alone (Table 1). For example, the excess ^210^Pb inventory is 61,300 ± 1,700 Bq · m^−2^, which leads to a mean annual flux (Eq. 2) of 1,914 ± 54 Bq · m^−2^ · yr^−1^. This value is 20 times higher than the flux observed for Darwin, Australia^20^. The other radionuclide inventories (^239^Pu, ^240^Pu and ^241^Am) are also high. This strongly suggests that sediment focusing is occurring at the study site, which enables study of deposition conditions with high time resolution. This finding is in agreement with the high accumulation rates observed at the study site, which are exceptional for deep sea sediments.

In contrast, the observed inventory for ^137^Cs of 84 ± 15 Bq · m^−2^ (September 2005) is lower than what would be expected to be deposited at this site from global fallout. A reference value to compare the ^137^Cs inventory to is about 250 Bq · m^−2^ as a mean value for 0–10°S latitude band, decay corrected to the year 2005^4^. A possible explanation for this observed mismatch in inventories is the different biochemical behaviour of caesium, which is rather soluble in the marine environment. Plutonium and americium, being particle reactive, are more rapidly scavenged in areas with sufficient particle availability^11^.

The ^240^Pu/^239^Pu atom ratio can provide information about the origin of plutonium in the environment. Early atmospheric testing in the years 1952–1954 was dominated by high ^240^Pu/^239^Pu ratio U.S. tests at the Marshall Islands. In October 1952 the first thermonuclear test, Ivy Mike, was conducted at Enewetak Atoll. Airborne and condensed debris from the test yielded a ^240^Pu/^239^Pu atom ratio of 0.363 ± 0.004^21^. A recent high resolution study of plutonium accumulation history in a coral from Enewetak Atoll lagoon provides refined information about Pu ratios in the local PPG fallout^22^. This uncovered a wide variation in ^240^Pu/^239^Pu atom ratios with time; during the period 1952–1955 the values decreased from 0.424 ± 0.026 to 0.234 ± 0.016. The value related to the largest test, Ivy Mike, was found to be very close to that previously reported: 0.379 ± 0.038^22^. Furthermore, the largest U.S. thermonuclear test at Bikini Atoll, Castle Bravo in March 1954, heavily contaminated the atoll with high (>0.3) ^240^Pu/^239^Pu atom ratio fallout^5^,^23^.

In the time period between 1955 and 1958 the U.S. tests continued to produce high ^240^Pu/^239^Pu ratio material^24^, however a number of smaller tests yielded local fallout with much lower ratios^22^. For instance, tests at Enewetak Atoll (e.g., Redwing Apache in 1956 or Hardtack I Oak in 1958) were characterized by ^240^Pu/^239^Pu atom ratios of ~0.17 and ~0.08, respectively^22^. A substantial contribution to the total explosive yield in this period also came from tests conducted by the U.S.S.R. and the U.K., and overall there was a general decrease in the isotopic ratio to ~0.22, recorded in Arctic and Antarctic glaciers^25^.

Atmospheric testing was briefly interrupted worldwide during the three year moratorium (1958–1961). During the post-moratorium atmospheric testing period in 1961–1963 the total yield peaked^25^ with a series of very large tests by the U.S.S.R. at Novaya Zemlya, and the isotopic ratio decreased further. The Partial Nuclear Test Ban Treaty came into effect in October 1963 leading to a subsequent significant decrease in atmospheric fallout. While French and Chinese atmospheric tests continued until the 1980’s, these were of much lower yield than the earlier tests of the U.S.A. and U.S.S.R. Although the atmospheric radionuclide concentrations decreased, radionuclides in the oceans remain to great part in the water column.

The fraction of PPG derived plutonium in the studied core was calculated from the PPG and global fallout end-members (Eqs 4 and 5). In previous studies, values of 0.36 or 0.30 were used as a PPG end-member for distinguishing between different plutonium sources^6^,^10^,^12^,^13^,^26^. In the light of the latest results^22^, we consider the value 0.36 as one representing well the earliest testing period at the PPG. For the younger sediment core section, fractions of PPG plutonium calculated using this ratio might be underestimated. For more recent PPG derived plutonium, we have used an alternative end-member value of 0.27 ± 0.03, to better reflect the material being continuously released from the PPG sediments. This ratio represents an average value from a coral-based record of plutonium deposition at the Guam Island between years 1981 and 1999^27^. The PPG fraction quantified using this ratio might well represent the upper estimate. A ^240^Pu/^239^Pu atom ratio of 0.176 ± 0.008, calculated from 15 soil samples collected between 30°N and 30°S^28^, is considered characteristic of integrated global fallout and this value was used as the global fallout end-member in the mixing model.

**Figure 4:**
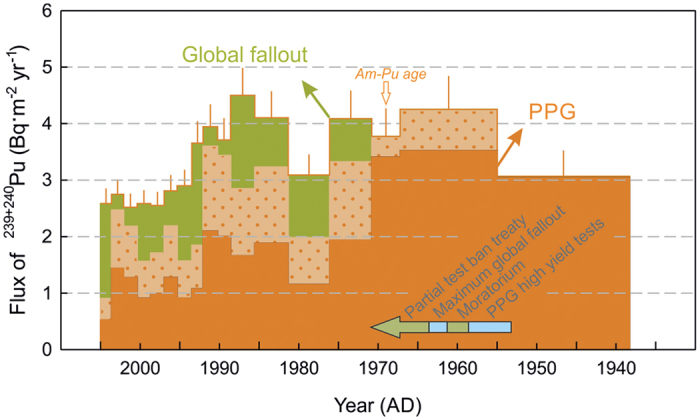
The time-line of combined ^239^Pu and ^240^Pu fluxes to the Lombok Basin sediments. The temporal axis was derived from the ^210^Pb age model. The minimum fraction of PPG derived plutonium for each analysed sediment core interval is distinguished by the dark orange colour (derived from Model 1), while the dotted area shows the limit of PPG derived fraction (calculated using Model 2). The remaining fraction in green represents the global fallout plutonium. The error bars (1 *σ*) consist of combined Pu activity and accumulation rate uncertainties.

The ^240^Pu/^239^Pu atom ratios (Fig. 3d) and calculated fractions (Supplementary Table S2) show, that virtually all of plutonium in the deepest sediment slices of our core can be assigned to be of PPG origin. In Fig. 4 combined ^239+240^Pu yearly depositions over the studied time interval together with PPG fraction estimates are displayed. The first arrival of PPG radionuclide enriched waters could be expected after 1952: a high resolution ^14^C coral record from the Makassar Strait^15^ shows elevated ^14^C in 1955 following the Castle Bravo test. This is in a good agreement with the observed appearance of Pu and Am in our sediment core within the period of the first large tests; the bottom and top of the first Pu-rich layer in the sediment core are dated at 1938 ± 6 and 1955 ± 6 AD, respectively.

The ^240^Pu/^239^Pu atom ratio and fractions derived from PPG versus integrated global fallout remain at elevated, rather constant levels during 1960’s. The plutonium isotope ratio is well above that recognised value for global fallout and strongly suggests that the material is originating almost exclusively from the PPG. Above 18 cm depth the ^240^Pu/^239^Pu atom ratio decreases significantly from ~0.34 to ~0.24, presumably largely due to the influence of the post-moratorium tests at Novaya Zemlya leading to global fallout with a lower ratio.

In the upper part of the core covering the 1970’s to the 2000’s, approximately 30–60% can be attributed to integrated global fallout. The remaining portion is likely transported from the PPG as a result of remobilization of plutonium deposited from the close-in fallout in the Marshall Islands area. The plutonium flux to the sediment remains high (2.5–4.5 Bq · m^−2^yr^−1^) over the entire 1950’s–2000’s period. The slow decrease in the transuranic isotope deposition rates is especially remarkable, because it contrasts the much more rapid decrease observed in high resolution coral archives at multiple Northwest Pacific sites^22^,^27^,^29^. The biogeochemical reasons for this mismatch deserve further investigation.

Activity ratios of ^241^Am/^239^Pu increase in the upper part of the core, from around 2.5 at 30 cm depth to close to 4 towards the surface (Fig. 3c). This increase is attributed to the steady increase in the proportion of Am to Pu, as a consequence of radioactive decay and the relatively short 14.35 year half-life of ^241^Pu, that occurs in the PPG sediments and also during transport to the study site, combined with different behaviour of the two elements during re-mobilisation (from the sediment back into the water column at the PPG), transport and subsequent scavenging processes prior to deposition in the Lombok Basin.

The age of the Pu source term was calculated using the measured activities of ^241^Pu and ^241^Am for the 18–20 cm depth slice. The ^210^Pb dates of the bottom and top of the sediment core interval are 1967 ± 5 and 1970 ± 4, respectively, and the ^240^Pu/^239^Pu atom ratio indicates a minimum of 90% of PPG derived plutonium. Using Eq. 7 and the ^241^Pu – ^241^Am data from this slice, the plutonium age^6^,^30^ was determined as 1945 ± 5. This apparent age is older than 1952 Ivy Mike test, possibly a result of preferential transport and/or scavenging of americium over plutonium, as indicated by the observed increase in the ^241^Am/^239^Pu ratio noted above, and as documented for instance in the Atlantic Ocean^31^ and the Mediterranean Sea^32^.

Uranium ions have shown to be strongly conserved in seawater^33^ and anthropogenic ^236^U has recently seen interest as a conservative oceanic tracer. Currently there are few studies confirming negligible scavenging of anthropogenic ^236^U to the seabed^34^. The absence of ^236^U in our sediment with high concentrations of other anthropogenic radionuclides supports the suggestion of this isotope being a suitable conservative oceanic tracer.

In summary, our study has shown that the previously observed high ^241^Am concentrations at the Lombok Basin site are correlated with similarly high concentrations of plutonium isotopes. Our results demonstrate that the transport of the transuranic radioisotopes through the ITF continues to this day, and that over the period since the cessation of atmospheric nuclear weapons testing, the rate of their deposition to the sediments has decreased more slowly than would be expected from coral records. Based on the end-member analysis of plutonium atom ratios we infer that the portion of PPG derived plutonium was high until 1970’s and remains significant.

## Methods

### Sediment coring

Sediment core GeoB10065–9 was taken with a multicorer (MUC) on 5. September 2005 during the R/V SONNE SO-184 cruise in the Lombok Basin (9°13.41′ 118°53,55′, 1,284 m, length 50 cm). The material used for the analysis within this study is identical to the sediment core described as MUC-B previously^17^. The inner diameter of the transparent sampling tube was 10 cm. The core was recovered with a visibly undisturbed sediment - water interface.

### Gamma spectrometry

The sediment samples were measured freeze dried. Material was taken in 1 cm slices between 0–10 cm, 2 cm slices between 10–20 cm and 5 cm slices between 20–50 cm core depth; a total number of 21 samples was measured. Individual sample masses varied between 1–3 g. Transparent polystyrene cylindrical dishes with an inner diameter of 34.7 mm and height of 12.6 mm were filled with the samples to heights of 2–5 mm. All samples in their containers were sealed by welding using a gas tight foil and the measurement was started more than 3 weeks after sealing, in order to ensure, that the radioactive equilibrium between ^226^Ra, ^222^Rn and its progeny had been established.

A coaxial HPGe detector (Canberra Industries, 50% rel. efficiency) housed in 10 cm Pb shielding with Cu, Cd and plastic linings operated under Genie 2000 software was used for gamma spectrometry. The detection efficiencies were calculated using LabSOCS software (Canberra) and verified using measurements of certified materials in similar geometries. The overall uncertainties combine errors arising from counting statistics, efficiency calibration, sample mass determination and decay data. More details on analytical uncertainties are available in the Supplementary material.

### Radiochemical extraction

The method is based on Srncik *et al*.^35^ with minor modifications which are described briefly. The samples (1–3 g) were ashed at 450 °C and then spiked with ~10^10^ atoms each of ^242^Pu (SRM 4334H, NIST), ^233^U (CRM 111-A, NBL) and ^243^Am (SRM 4332C, NIST) in solutions derived from the given standards. After leaching in 8 M HNO_3_ the undissolved residues were filtered and about 0.2 g NaNO_2_ was added to adjust the oxidation state of Pu to Pu(IV).

### Am separation from Pu and U

A column filled with 0.5 g UTEVA^©^ (100–150 *μ*m) was mounted underneath a column filled with 2 g of BioRad AG^©^ 1 × 8 (100–200 mesh, Cl^−^ form). After conditioning with 8 M HNO_3_ the samples were transferred onto the resins followed by rinses with 8 M HNO_3_ and 4 M HNO_3_. Then the columns were separated and all transmitted solutions were retained as these contain the Am. 37% HCl was added to the BioRad AG^©^ 1 × 8 resins to remove Th, and Pu was then eluted with 0.1 M NH_4_I-9M HCl. 6 M HCl was added to the UTEVA^©^ resins prior the U elution with 1 M HCl. Both the Pu and U fractions were evaporated and 1.5 mg Fe was added as FeCl_3_.

### Am purification

The Am fractions were evaporated, dissolved in 2 M HNO_3_ and few grains of NH_4_SCN were added to indicate the presence of iron which was then reduced with ascorbic acid to Fe(II). After conditioning 0.74 g TRU^©^ (100–150 *μ*m) resin per sample with 2 M HNO_3_ the samples were loaded onto the columns, followed by washes with 2 M HNO_3_ and 9 M HCl before Am was eluted with 4 M HCl. The Am fractions were evaporated and 1.5 mg Fe was added.

### Final preparation for AMS measurement

The Pu, U and Am fractions were dried for at least 48 h at 90 °C and then combusted at 800 °C for 8 h. The Pu and Am fractions were mixed with Ag powder (4x the sample weight), while the U samples were mixed with 1 mg of Al powder. Each mixture was then pressed into individual aluminium sample holders for the AMS measurement. The Ag and Al powders are added to ensure electrical and thermal conductivity.

### Accelerator mass spectrometry

Isotope concentrations were determined with AMS using the 14UD pelletron accelerator at the ANU based on the methods described in Fifield^36^. The method provided above effectively separates americium, plutonium and uranium, however the ^236^U content in these sediment samples was below our detection limit, and results for only americium and plutonium isotopes are reported.

Negatively-charged monoxide ions from the purified Am (Pu) samples were extracted using a Cs sputter ion source. The ions were accelerated to 105 keV, mass-selected with a high resolution (M/ΔM ≥ 300) magnet, focussed and injected into the 14UD accelerator, which was operated at ~4 MV. A low-pressure gas stripper in the high-voltage terminal at the centre of the accelerator dissociates the 4 MeV molecular ions and removes outer electrons from the atom species. The positively charged atoms were then again accelerated and Am^5+^ (Pu^5+^) ions of energy ~24 MeV selected by an analysing magnet.

The isotopes were measured using a slow cycling sequence to determine the relative count rates for each isotope. A propane-filled ionisation chamber was used to identify and count individual atoms. Measurement times for americium isotopes were typically 1 min for ^243^Am and 2 min for ^241^Am, and for the plutonium isotopes 1 min for ^242^Pu, 2 min for ^239^Pu and 3 min for ^240^Pu. At least two cycles of the sequence, and concluding with a ^243^Am (^242^Pu) measurement, made for each sample. ^241^Am (^239,240^Pu) concentrations were deduced from the known mass of the ^243^Am (^242^Pu) spike added to the sample.

Analytical errors are quantified in the Supplementary material.

### Age model

The chronology of the sediment core was constructed using the constant rate of supply (CRS) model^37^, assuming constant flux of ^210^Pb to the sediment. The chronology data are provided in Supplementary Table S1.

### Inventories and fluxes

The radionuclide inventories (Bq · m^−2^) were calculated as a sum of measured values of activity concentration *C*_*i*_ in the sediment core as:


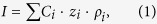


where *z*_*i*_ is the thickness and *ρ*_*i*_ the dry bulk density of the *i*^*th*^ core slice. If a constant flux of the radionuclide is presumed, the average yearly flux (Bq · m^−2^ · yr^−1^) can be calculated as:


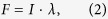


where *λ* (yr^−1^) is the radionuclide’s decay constant.

Dates from the CRS age model allow for the determination of a mass accumulation rate, *r*_*i*_ (g · m^2^yr^−1^), for each core slice *i*. We have used these to determine the associated plutonium fluxes, *F*_*i*_, viz:


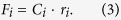


### Source attribution

Assuming that the plutonium activity in the core is a mixture of Global fallout and PPG,


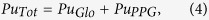


the contributions of global and PPG derived plutonium was resolved from the measured ^240^Pu/^239^Pu atom ratios *R*_*M*_ using a two end-member mixing model^38^:


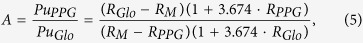


where 3.674 is a factor for conversion between atom and activity ratios of ^240^Pu/^239^Pu. Two scenarios were considered to estimate both the initial and ongoing PPG fractions:

- Model 1: *R*_*Glo*_ = 0.18 ± 0.01 for integrated global fallout^28^, and *R*_*PPG*_ = 0.36 ± 0.02 for PPG^21^,
- Model 2: *R*_*Glo*_ = 0.18 ± 0.01 for global fallout end-member^28^, and *R*_*PPG*_ = 0.27 ± 0.03 for PPG^27^.

The portions of activity due to PPG from total Pu activities are then calculated by solving the system of Eqs 4 and 5:


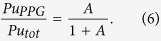


### Am-Pu age

The age of the Pu source term was calculated using the measured activities of ^241^Pu and ^241^Am,


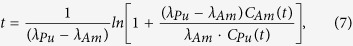


assuming that initial ^241^Am = 0. *λ*_*Pu*_ and *λ*_*Am*_ are decay constants of ^241^Pu and ^241^Am, respectively.

## Additional Information

**How to cite this article:** Pittauer, D. *et al*. Continuous transport of Pacific-derived anthropogenic radionuclides towards the Indian Ocean. *Sci. Rep.* **7**, 44679; doi: 10.1038/srep44679 (2017).

**Publisher's note:** Springer Nature remains neutral with regard to jurisdictional claims in published maps and institutional affiliations.

## Supplementary Material

Supplementary Material

## Figures and Tables

**Figure 1:**
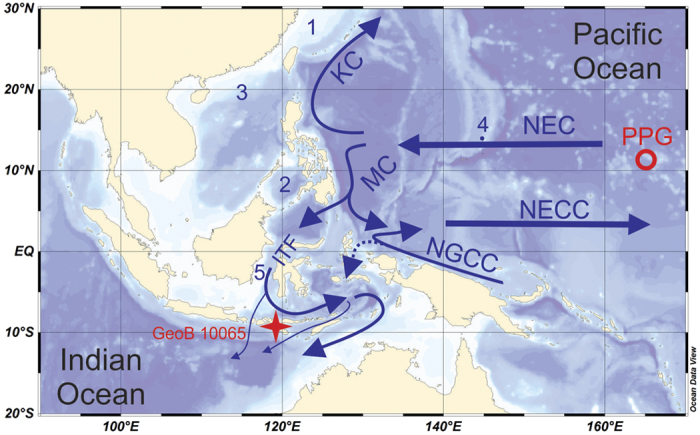
The location of the study site GeoB 10065, marked by a star, within the Indonesian Throughflow (ITF). Major ocean currents relevant for the Pacific - Indian Ocean water exchange are displayed schematically with arrows: North Equatorial Current (NEC) bifurcating into Kuroshio Current (KC) and Mindanao Current (MC), North Equatorial Countercurrent (NECC), New Guinea Coastal Current (NGCC). The U.S. nuclear test site Pacific Proving Grounds (PPG) is marked by a circle. Other locations mentioned in the text are marked by numbers: 1 East China Sea, 2 Sulu Sea, 3 South China Sea, 4 Island of Guam and 5 Makassar Strait. This map was created by using Ocean Data View software^39^.

**Table 1:** Radionuclide inventories measured at the study site.

Inventory	Bq · m^−2^	Remarks
Excess ^210^Pb	61,300 ± 1,700^*^	Expected: 3,040 (Darwin, Australia)^20^
^137^Cs	84 ± 15^*^	Expected: 250^*^ (0–10°S)^4^
^239^Pu	106.9 ± 1.6	
^240^Pu	112.3 ± 2.2	
^241^Am	314 ± 18^**^	

*Decay corrected to the sampling date: September 2005.

**Reported to the measurement date: July 2015.

## References

[b1] Lindahl P., Lee S.-H., Worsfold P. & Keith-Roach M. Plutonium isotopes as tracers for ocean processes: A review. Marine Environmental Research 69, 73–84 (2010). 19773079 10.1016/j.marenvres.2009.08.002

[b2] Hamilton T. Linking legacies of the cold war to arrival of anthropogenic radionuclides in the oceans through the 20^*th*^ century. In Livingston H. D. (ed.) Marine Radioactivity vol. 6 of Radioactivity in the Environment, 23–78 (Elsevier, 2005).

[b3] Vintró L. L., Mitchell P. I., Smith K. J., Kershaw P. J. & Livingston H. D. Transuranium nuclides in the world’s oceans. In Livingston H. D. (ed.) Marine Radioactivity, vol. 6 of Radioactivity in the Environment, 79–108 (Elsevier, 2005).

[b4] UNSCEAR. Report to the General Assembly, with scientific annexes. Sources and effects of ionizing radiation. Volume 1: Sources. Annex C, United Nations Scientific Committee on the Effects of Atomic Radiation, New York (2000).

[b5] Muramatsu Y. . Measurement of ^240^Pu/^239^Pu isotopic ratios in soils from the Marshall Islands using ICP-MS. Science of the Total Environment 278, 151–159 (2001). 11669263 10.1016/s0048-9697(01)00644-1

[b6] Lee S.-H. . Distribution and inventories of ^90^Sr, ^137^Cs, ^241^Am and Pu isotopes in sediments of the Northwest Pacific Ocean. Marine Geology 216, 249–263 (2005).

[b7] Moon D.-S. . Accumulation of anthropogenic and natural radionuclides in bottom sediments of the Northwest Pacific Ocean. Deep Sea Research Part II: Topical Studies in Oceanography 50, 2649–2673 (2003).

[b8] Andrews A. H., Asami R., Iryu Y., Kobayashi D. R. & Camacho F. Bomb-produced radiocarbon in the western tropical Pacific Ocean: Guam coral reveals operation-specific signals from the Pacific Proving Grounds. Journal of Geophysical Research: Oceans 121, 6351–6366 (2016).

[b9] Tims S. . Plutonium AMS measurements in Yangtze River estuary sediment. Nuclear Instruments and Methods in Physics Research Section B: Beam Interactions with Materials and Atoms 268, 1155–1158 (2010).

[b10] Wang Z.-l. & Yamada M. Plutonium activities and ^240^Pu/^239^Pu atom ratios in sediment cores from the east China sea and Okinawa Trough: Sources and inventories. Earth and Planetary Science Letters 233, 441–453 (2005).

[b11] Lee S.-Y., Huh C.-A., Su C.-C. & You C.-F. Sedimentation in the Southern Okinawa Trough: enhanced particle scavenging and teleconnection between the Equatorial Pacific and western Pacific margins. Deep Sea Research Part I: Oceanographic Research Papers 51, 1769–1780 (2004).

[b12] Zheng J. & Yamada M. Sediment core record of global fallout and Bikini close-in fallout Pu in Sagami Bay, Western Northwest Pacific margin. Environmental Science and Technology 38, 3498–3504 (2004). 15296298 10.1021/es035193f

[b13] Dong W., Zheng J., Guo Q., Yamada M. & Pan S. Characterization of plutonium in deep-sea sediments of the Sulu and South China Seas. Journal of Environmental Radioactivity 101, 622–629 (2010). 20403651 10.1016/j.jenvrad.2010.03.011

[b14] Wu J. . Isotopic composition and distribution of plutonium in northern South China Sea sediments revealed continuous release and transport of Pu from the Marshall Islands. Environmental Science and Technology 48, 3136–3144 (2014). 24564849 10.1021/es405363q

[b15] Fallon S. & Guilderson T. Surface water processes in the Indonesian throughflow as documented by a high-resolution coral Δ^14^C record. Journal of Geophysical Research: Oceans 113 (2008).

[b16] Yamada M., Zheng J. & Wang Z.-L. ^137^Cs, ^239+240^Pu and ^240^Pu/^239^Pu atom ratios in the surface waters of the western North Pacific Ocean, eastern Indian Ocean and their adjacent seas. Science of The Total Environment 366, 242–252 (2006). 16165190 10.1016/j.scitotenv.2005.08.014

[b17] Steinke S. . Mid- to late-holocene Australian-Indonesian summer monsoon variability. Quaternary Science Reviews 93, 142–154 (2014).

[b18] Baumgart A., Jennerjahn T., Mohtadi M. & Hebbeln D. Distribution and burial of organic carbon in sediments from the Indian Ocean upwelling region off Java and Sumatra, Indonesia. Deep Sea Research Part I: Oceanographic Research Papers 57, 458–467 (2010).

[b19] Gingele F. X., Deckker P. D. & Hillenbrand C.-D. Clay mineral distribution in surface sediments between Indonesia and NW Australia – source and transport by ocean currents. Marine Geology 179, 135–146 (2001).

[b20] Preiss N., Mélières M.-A. & Pourchet M. A compilation of data on lead 210 concentration in surface air and fluxes at the air-surface and water-sediment interfaces. Journal of Geophysical Research 101, 28847–28862 (1996).

[b21] Diamond H. . Heavy isotope abundances in Mike thermonuclear device. Physical Review 119, 2000–2004 (1960).

[b22] Froehlich M., Chan W., Tims S., Fallon S. & Fifield L. Time-resolved record of ^236^U and ^239,240^Pu isotopes from a coral growing during the nuclear testing program at Enewetak Atoll (Marshall Islands). Journal of Environmental Radioactivity 165, 197–05 (2016). 27764678 10.1016/j.jenvrad.2016.09.015

[b23] Komura K., Sakanoue M. & Yamamoto M. Determintion of Pu-240/Pu-239 ratio in environmental samples based on the measurements of Lx/Alpha-ray activity ratio. Health Physics 46, 1213–1219 (1984). 6724934 10.1097/00004032-198406000-00005

[b24] Noshkin V. E., Wong K. M., Eagle R. J. & Gatrousis C. Transuranics and other radionuclides in Bikini Lagoon: Concentration data retrieved from aged coral sections. Limnology and Oceanography 20, 729–742 (1975).

[b25] Koide M., Bertine K., Chow T. J. & Goldberg E. D. The ^240^Pu/^2390^Pu ratio, a potential geochronometer. Earth and Planetary Science Letters 72, 1–8 (1985).

[b26] Pan S., Tims S., Liu X. & Fifield L. ^137^Cs, ^239+240^Pu concentrations and the ^240^Pu/^239^Pu atom ratio in a sediment core from the sub-aqueous delta of Yangtze River estuary. Journal of Environmental Radioactivity 102, 930–936 (2011). 20561723 10.1016/j.jenvrad.2010.05.012

[b27] Lindahl P. . Sources of plutonium to the tropical Northwest Pacific Ocean (1943–1999) identified using a natural coral archive. Geochimica et Cosmochimica Acta 75, 1346–1356 (2011).

[b28] Kelley J., Bond L. & Beasley T. Global distribution of Pu isotopes and ^237^Np. Science of the Total Environment 237–238, 483–500 (1999). 10.1016/s0048-9697(99)00160-6 10568297

[b29] Lindahl P. . Spatial and temporal distribution of Pu in the Northwest Pacific Ocean using modern coral archives. Environment International 40, 196–201 (2012). 21890207 10.1016/j.envint.2011.08.004

[b30] Smith J., Ellis K., Naes K., Dahle S. & Matishov D. Sedimentation and mixing rates of radionuclides in Barents Sea sediments off Novaya Zemlya. Deep Sea Research Part II: Topical Studies in Oceanography 42, 1471–1493 (1995).

[b31] Cochran J. K., Livingston H. D., Hirschberg D. J. & Surprenant L. D. Natural and anthropogenic radionuclide distributions in the northwest Atlantic Ocean. Earth and Planetary Science Letters 84, 135–152 (1987).

[b32] Lee S.-H. . Recent inputs and budgets of ^90^Sr, ^137^Cs, ^239,240^Pu and ^241^Am in the northwest Mediterranean Sea. Deep Sea Research Part II: Topical Studies in Oceanography 50, 2817–2834 (2003).

[b33] Dunk R., Mills R. & Jenkins W. A reevaluation of the oceanic uranium budget for the Holocene. Chemical Geology 190, 45–67 (2002).

[b34] Sakaguchi A. . Uranium-236 as a new oceanic tracer: A first depth profile in the Japan Sea and comparison with caesium-137. Earth and Planetary Science Letters 333–334, 165–170 (2012). 10.1016/j.epsl.2012.04.004 PMC3617607 23564965

[b35] Srncik M., Hrnecek E., Steier P. & Wallner G. Determination of U, Pu and Am isotopes in Irish Sea sediment by a combination of AMS and radiometric methods. Journal of Environmental Radioactivity 102, 331–335 (2011). 21316820 10.1016/j.jenvrad.2011.01.004

[b36] Fifield L. Accelerator mass spectrometry of the actinides. Quaternary Geochronology 3, 276–290 (2008).

[b37] Appleby P. G. & Oldfield F. The calculation of lead-210 dates assuming a constant rate of supply of unsupported ^210^Pb to the sediment. CATENA 5, 1–8 (1978).

[b38] Krey P. W. Remote plutonium contamination and total inventories from Rocky Flats. Health Physics 30, 209–214 (1976). 1245421 10.1097/00004032-197602000-00009

[b39] Schlitzer R. Ocean Data View. http://odv.awi.de (2016).

