# Supplementary material for: Continuous transport of Pacific-derived anthropogenic radionuclides towards the Indian Ocean
Source: Sci Rep. 2017 Mar 17;7:44679. doi: 10.1038/srep44679 (PMC5356341; doi:10.1038/srep44679)
Supplement: Supplementary Material [file srep44679-s1.pdf]

Supplementary material for: Continuous transport of Pacific-derived anthropogenic radionuclides towards the Indian Ocean

Daniela Pittauer[1, 2, *], Stephen G. Tims[3], Michaela B. Froehlich[3], L. Keith Fifield[3], Anton Wallner[3], Steven D. McNeil[3], and Helmut W. Fischer[2]

[1]University of Bremen, MARUM - Center for Marine Environmental Sciences, Bremen, 28359, Germany
[2]University of Bremen, Institute of Environmental Physics, Bremen, 28359, Germany
[3]Australian National University, Department of Nuclear Physics, Canberra, ACT 2601, Australia
[*]pitdana@yahoo.co.uk

ABSTRACT

This file provides supplementary information to the manuscript "Continuous transport of Pacific-derived anthropogenic radionuclides towards the Indian Ocean". The data are additionally stored at the digital data library PANGAEA (https://doi.pangaea.de/10.1594/PANGAEA.871726).

Analytical uncertainties

Analytical uncertainties in gamma spectrometry

The uncertainty budget comprises of systematic and counting errors. The main constituent for systematic uncertainties is the sourceless efficiency calibration performed by LabSOCS for a characterized detector produced by Canberra. These uncertainties range according to manufacturer's recommendation from 10% for gamma energies below 150 keV to 4% for energies above 1,000 keV.

The counting errors for radionuclides in sediment samples in this measurement setup vary mainly depending on activities in samples, as well as counting times (41–114 hours) and sample masses (1.05–3.05 g dry mass). For ^{210}Pb counting errors are typically $<$5%, increasing with decreasing ^{210}Pb activity. Counting errors for radon daughter nuclides used for ^{226}Ra quantification decrease with increasing measurement time and sample mass. For individual gamma lines of ^{214}Pb and ^{214}Bi, the counting errors are, on average, 7.1% for 352 keV, 8.2% 609 keV, 11.0% for 295 keV, 12.0% for 242 keV, 13.7% for 1764 keV and 21.7% for 1120 keV. The counting errors for ^{241}Am are high, 44% on average, mainly due to high spectrum continuum in low energies. ^{137}Cs, which was not detectable in individual spectra, could be detected in summed up spectra of 5 successive 1 cm depth intervals with high counting errors ($>$30%).

Analytical uncertainties in accelerator mass spectrometry

The statistical errors on the ^{239}Pu, ^{241}Pu and ^{241}Am measurements were typically $\sim$2.5%, $\sim$13% and $\sim$6%, respectively. The statistical error on the ^{240}Pu/^{239}Pu ratio was $\sim$5%. Total systematic errors for the AMS measurements was $\sim$4%, with the exception of that for ^{241}Am. The total systematic error for ^{241}Am was $\sim$18.5%, which comprised a 4% component arising from the measurement technique and an 18% error component from the calibration on the ^{243}Am spike.

Sediment core chronology

The activity concentrations of excess ^{210}Pb (^{210}Pb$_{xs}$)[1] in the multicorer core GeoB10065-9 MUC-B were used for sediment chronology within this study (Tab. S1). ^{210}Pb$_{xs}$ was quantified as a difference between measured total ^{210}Pb (^{210}Pb$_{tot}$) and supported ^{210}Pb (^{210}Pb$_{sup}$). ^{210}Pb$_{sup}$ was determined for each sample individually as a mean value of ^{214}Pb and ^{214}Bi (quantified via 242, 295 and 352 keV and 609, 1120 and 1764 keV lines, respectively, as a weighted average activity from multiple energies). ^{214}Pb and ^{214}Bi are assumed to be in radioactive equilibrium with ^{226}Ra. The average value of ^{210}Pb$_{sup}$ throughout the core was 62.9 ± 7.6 Bq·kg^{-1} d.m., which is less than 5% of ^{210}Pb$_{tot}$ in the top part of the core.

The constant rate of supply model (CRS)[2,3] was used for calculation of the individual sediment layers' ages, as well as sedimentation and accumulation rates, and their uncertainties.[3] Dry bulk density (DBD) was used for correction of compaction effect. As the DBD was not measured directly on the MUC material, we used an extrapolation from the data measured on the parallel gravity core GeoB10065-7[4] including a depth offset of 20 cm (Fig. S1), which were presumably lost during the sampling with the gravity corer. The extrapolation to the upper 50 cm of the MUC was performed using a polynomial function of the 5th order $DBD = 7.425 \cdot 10^{-15} Z^5 - 2.108 \cdot 10^{-11} Z^4 + 2.296 \cdot 10^{-8} Z^3 - 1.185 \cdot 10^{-5} Z^2 + 2.922 \cdot 10^{-3} Z + 0.2121$, where Z is depth (cm). This fit follows the data closely, although it does not have any physical meaning.

The activities at the base of the core do not converge to "zero" (the dating horizon has not been reached).[5] In order to avoid underestimation of inventories and therefore overestimation of ages in the deeper layers,[6] the residual inventory of 560±100 $Bq \cdot kg^{-1}$ was added to the total inventory. It was estimated as an integral below an exponential function derived from the fit through the $^{210}Pb_{xs}$ activity concentrations vs mass depth in the interval 15–50 cm. This is less than 1% of the total inventory value of 61,300 $Bq \cdot m^{-2}$.

Artificial radionuclides

Artificial radionuclide concentrations and calculated fractions of PPG fallout (for two different end-members) are listed in Tab. S2. The uncertainties of PPG fractions were calculated using the Monte Carlo method in R.[7]

References

1. Steinke, S. *et al.* Mid- to late-holocene Australian-Indonesian summer monsoon variability. *Quaternary Science Reviews* **93**, 142–154 (2014).
2. Appleby, P. G. & Oldfield, F. The calculation of lead-210 dates assuming a constant rate of supply of unsupported ^{210}Pb to the sediment. *CATENA* **5**, 1–8 (1978).
3. Sanchez-Cabeza, J. & Ruiz-Fernández, A. ^{210}Pb sediment radiochronology: An integrated formulation and classification of dating models. *Geochimica et Cosmochimica Acta* **82**, 183–200 (2012).
4. Mohtadi, M. Dry bulk density of sediment core GeoB10065-7. Published in the digital data library PANGAEA, https://doi.pangaea.de/10.1594/PANGAEA.872006 (2017).
5. Appleby, P. G. Dating recent sediments by ^{210}Pb: Problems and solutions. Proc., 2nd NKS/EKO-1 Seminar, Helsinki, 2-4 April 1997, STUK, Helsinki (1998).
6. MacKenzie, A., Hardie, S., Farmer, J., Eades, L. & Pulford, I. Analytical and sampling constraints in ^{210}Pb dating. *Science of The Total Environment* **409**, 1298–1304 (2011).
7. R Core Team. *R: A Language and Environment for Statistical Computing*. R Foundation for Statistical Computing, Vienna, Austria (2013). URL http://www.R-project.org/.

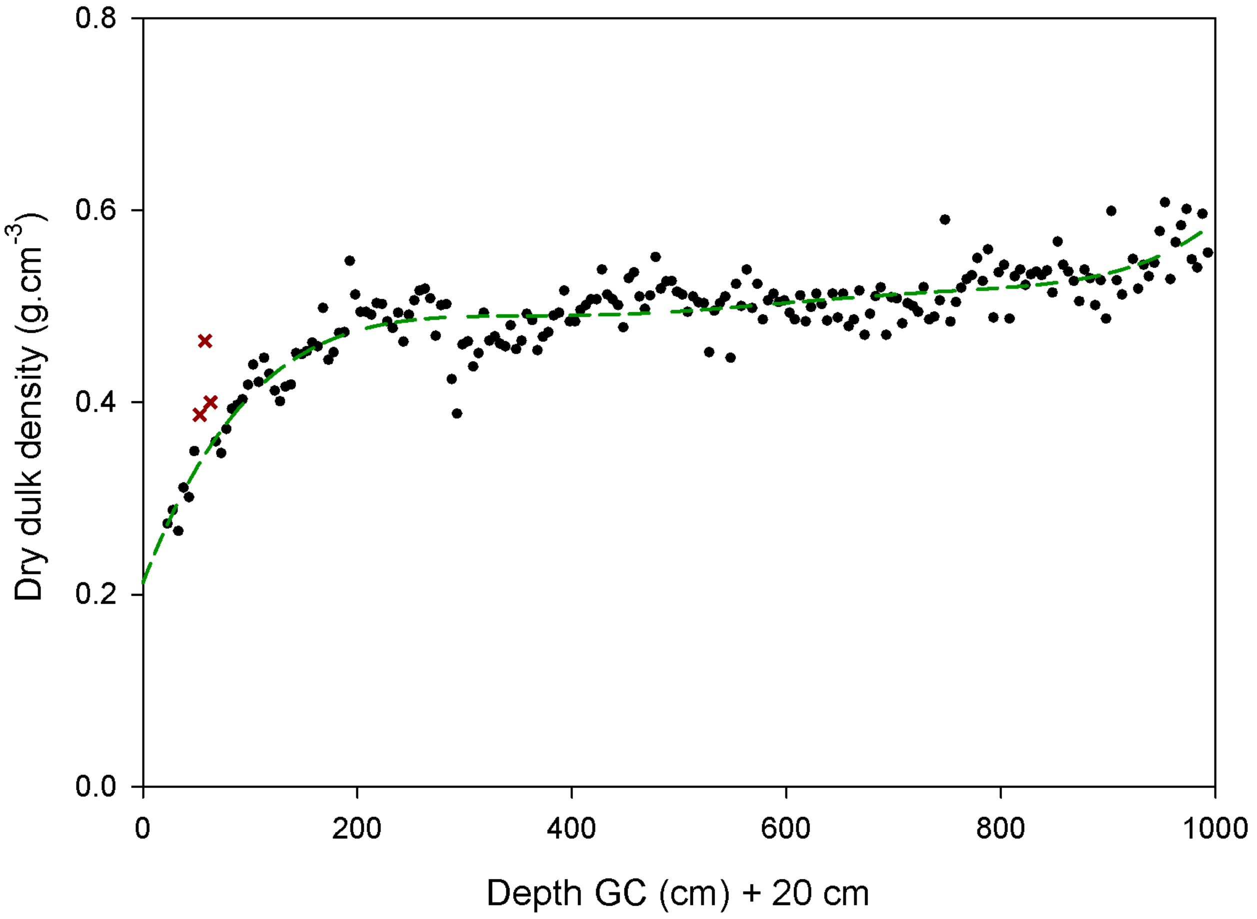

Figure S1. Dry bulk density[4] extrapolation at site GeoB10065. Three datapoints (crosses) were excluded from the fit - they represent a material identified as a turbidite.

Sampling interval cm	Mass depth g·cm^{-2}	C $^{210}Pb_{tot}$ Bq·kg^{-1}	C $^{210}Pb_{xs}$ Bq·kg^{-1}	Age (layer top) yr	Calendar year	Accumulation rate g·cm^{-2}yr^{-1}	Sedimentation rate cm·yr^{-1}
0 – 1	0.1	1297 ± 108	1224 ± 108	0.0 ± 0.0	2005	0.156 ± 0.020	0.73 ± 0.10
1 – 2	0.3	1312 ± 113	1257 ± 113	1.4 ± 0.7	2004	0.147 ± 0.017	0.68 ± 0.08
2 – 3	0.5	1392 ± 115	1321 ± 115	2.9 ± 1.0	2002	0.135 ± 0.016	0.62 ± 0.07
3 – 4	0.8	1299 ± 95	1232 ± 95	4.6 ± 1.3	2000	0.130 ± 0.015	0.58 ± 0.07
4 – 5	1.0	1216 ± 92	1163 ± 92	6.3 ± 1.5	1999	0.131 ± 0.016	0.58 ± 0.07
5 – 6	1.2	1118 ± 91	1041 ± 91	8.0 ± 1.7	1997	0.135 ± 0.017	0.59 ± 0.07
6 – 7	1.4	1182 ± 88	1109 ± 88	9.7 ± 1.8	1995	0.131 ± 0.017	0.57 ± 0.07
7 – 8	1.7	850 ± 66	790 ± 66	11.5 ± 2.0	1993	0.141 ± 0.018	0.60 ± 0.08
8 – 9	1.9	1015 ± 75	957 ± 75	12.9 ± 2.2	1992	0.146 ± 0.019	0.62 ± 0.08
9 – 10	2.1	861 ± 68	804 ± 68	14.8 ± 2.3	1990	0.137 ± 0.019	0.57 ± 0.08
10 – 12	2.5	758 ± 68	685 ± 68	16.4 ± 2.5	1989	0.154 ± 0.022	0.63 ± 0.09
12 – 14	3.0	899 ± 81	833 ± 80	19.4 ± 2.8	1986	0.137 ± 0.021	0.55 ± 0.08
14 – 16	3.5	919 ± 83	870 ± 83	23.7 ± 3.2	1981	0.107 ± 0.017	0.42 ± 0.07
16 – 18	4.0	797 ± 65	733 ± 65	28.9 ± 3.7	1976	0.097 ± 0.016	0.37 ± 0.06
18 – 20	4.5	486 ± 46	423 ± 46	34.2 ± 4.2	1971	0.114 ± 0.020	0.43 ± 0.08
20 – 25	5.5	500 ± 40	439 ± 40	37.8 ± 4.7	1967	0.136 ± 0.026	0.50 ± 0.09
25 – 30	6.9	425 ± 35	368 ± 34	50.0 ± 5.5	1955	0.100 ± 0.020	0.35 ± 0.07
30 – 35	8.3	271 ± 24	210 ± 24	66.7 ± 6.2	1938	0.083 ± 0.017	0.28 ± 0.06
35 – 40	9.8	199 ± 23	138 ± 23	83.5 ± 7.2	1922	0.081 ± 0.020	0.26 ± 0.06
40 – 45	11.4	155 ± 18	85 ± 18	103.7 ± 8.7	1901	0.068 ± 0.020	0.21 ± 0.06
45 – 50	13.0	85 ± 17	31 ± 17	130.0 ± 11.5	1875	0.109 ± 0.071	0.33 ± 0.22

Table S1. Radiometric data (reported per dry mass) of the sediment core GeoB 10065-9 MUC-B and sediment chronology. All values are reported with 1 σ uncertainty.

Sampling interval cm	C ^{241}Am[a] Bq·kg^{-1}	C ^{241}Am[b] Bq·kg^{-1}	C ^{239}Pu[b] Bq·kg^{-1}	C ^{240}Pu[b] Bq·kg^{-1}	^{240}Pu/^{239}Pu atom ratio	Fraction PPG (Model 1)	Fraction PPG (Model 2)	C ^{137}Cs[a] Bq·kg^{-1}
0 – 1	<3.12	3.36 ± 0.67	0.939 ± 0.049	0.717 ± 0.052	0.208 ± 0.014	0.20 ± 0.12	0.35 ± 0.24	
1 – 2	<3.25	3.47 ± 0.71	0.954 ± 0.054	0.909 ± 0.073	0.259 ± 0.020	0.52 ± 0.13	0.90 ± 0.38	
2 – 3	3.50 ± 2.00	4.02 ± 0.80	0.959 ± 0.053	0.902 ± 0.066	0.256 ± 0.018	0.51 ± 0.12	0.87 ± 0.35	
3 – 4	3.72 ± 1.57	3.73 ± 0.74	1.080 ± 0.049	0.914 ± 0.047	0.230 ± 0.008	0.35 ± 0.08	0.60 ± 0.23	
4 – 5	1.67 ± 1.60	3.28 ± 0.65	1.041 ± 0.054	0.905 ± 0.062	0.237 ± 0.014	0.39 ± 0.11	0.67 ± 0.29	
5 – 6	4.27 ± 1.42	4.33 ± 0.86	1.092 ± 0.063	0.991 ± 0.074	0.247 ± 0.018	0.45 ± 0.12	0.78 ± 0.33	
6 – 7	4.49 ± 1.69	4.27 ± 0.84	1.208 ± 0.060	0.997 ± 0.063	0.225 ± 0.012	0.32 ± 0.10	0.54 ± 0.24	
7 – 8	3.62 ± 1.57	3.92 ± 0.77	1.435 ± 0.088	1.167 ± 0.100	0.221 ± 0.018	0.29 ± 0.14	0.51 ± 0.30	
8 – 9	5.37 ± 1.73	4.75 ± 0.92	1.379 ± 0.065	1.320 ± 0.071	0.261 ± 0.010	0.53 ± 0.08	0.91 ± 0.33	
9 – 10	4.16 ± 1.67	5.63 ± 1.07	1.379 ± 0.069	1.328 ± 0.081	0.262 ± 0.013	0.54 ± 0.09	0.92 ± 0.34	
10 – 12	6.85 ± 3.10	5.48 ± 1.09	1.574 ± 0.076	1.347 ± 0.079	0.233 ± 0.011	0.37 ± 0.09	0.63 ± 2.25	
12 – 14	<3.60	4.70 ± 0.92	1.562 ± 0.081	1.423 ± 0.091	0.248 ± 0.014	0.46 ± 0.10	0.79 ± 0.31	
14 – 16	<3.39	4.68 ± 0.90	1.548 ± 0.076	1.330 ± 0.081	0.234 ± 0.012	0.37 ± 0.09	0.64 ± 0.26	
16 – 18	4.10 ± 1.75	5.85 ± 1.12	2.198 ± 0.105	2.022 ± 0.113	0.250 ± 0.011	0.47 ± 0.09	0.81 ± 0.30	
18 – 20	3.75 ± 1.78	3.94 ± 0.76	1.483 ± 0.074	1.833 ± 0.104	0.337 ± 0.015	0.90 ± 0.10	n.a.	
20 – 25	4.60 ± 0.74	3.60 ± 0.70	1.433 ± 0.070	1.682 ± 0.094	0.320 ± 0.014	0.83 ± 0.09	n.a.	
25 – 30	<2.66	3.31 ± 0.63	1.293 ± 0.053	1.789 ± 0.090	0.358 ± 0.017	0.99 ± 0.11	n.a.	
30 – 35	<1.06	0.04 ± 0.02	0.029 ± 0.012	0.020 ± 0.012	n.a.	n.a.	n.a.	
35 – 40	<1.40		0.0010 ± 0.0007					
0 – 5								0.42 ± 0.51 *
5 – 10								1.03 ± 0.51
10 – 15								1.38 ± 0.65
15 – 20								1.06 ± 0.46
20 – 25								1.67 ± 0.52
25 – 30								1.00 ± 0.27

[a] Analyzed by gamma spectrometry.
[b] Analyzed by AMS.
* This value was calculated from summed up spectra of depth interval 1–5 cm in GeoB 10065-9 MUC-B.

Table S2. Radionuclide activities (in dry mass) measured by gamma spectrometry and AMS in sediment core GeoB 10065-9 MUC-B. Fraction of PPG plutonium calculated from ^{240}Pu/^{239}Pu atom ratio for 2 different PPG end-members (Model 1 and Model 2). ^{137}Cs activities[1] (decay corrected to the sampling date, September 2005) was analyzed by gamma spectrometry in a parallel core GeoB 10065-9 MUC-A. All values reported with 1 σ uncertainty.
